# Correction to “Continuous Antisolvent Crystallization of Carbamazepine Dihydrate: Experiments and Modeling”

**DOI:** 10.1021/acs.iecr.5c01727

**Published:** 2025-05-20

**Authors:** Vaishnavi G. Honavar, Raj Wagh, Atul H. Bari, Ryan G. Ellis, Nandkishor K. Nere, Vivek V. Ranade

**Affiliations:** † Multiphase Reactors and Intensification Group, SSPC, Taighde Éireann − Research Ireland Centre for Pharmaceuticals, Bernal Institute, 8808 University of Limerick, Limerick V94T9PX, Ireland; ‡ 80493 Institute of Chemical Technology, Marathwada Campus, Jalna, 431203 Mumbai, India; § R&D Process Engineering, 359181 AbbVie Inc., North Chicago, Illinois 60064, United States

A Conflict of Interest statement is added.

We respectfully request that this correction be published to ensure full transparency and compliance with disclosure policies. No other changes to the manuscript are needed.

